# A Codon-Pair Bias Associated With Network Interactions in Influenza A, B, and C Genomes

**DOI:** 10.3389/fgene.2021.699141

**Published:** 2021-07-06

**Authors:** Ewan P. Plant, Zhiping Ye

**Affiliations:** Laboratory of Pediatric and Respiratory Viral Disease, Office of Vaccines Research and Review, CBER, FDA, Silver Spring, MD, United States

**Keywords:** influenza, codon deoptimization, attenuation, selective pressure, codon-pair, evolutionary bias

## Abstract

A new codon-pair bias present in the genomes of different types of influenza virus is described. Codons with fewer network interactions are more frequency paired together than other codon-pairs in influenza A, B, and C genomes. A shared feature among three different influenza types suggests an evolutionary bias. Codon-pair preference can affect both speed of protein translation and RNA structure. This newly identified bias may provide insight into drivers of virus evolution.

## Introduction

The limited size of viral genomes places constraints on the evolution that can occur without accruing deleterious mutations (Holmes, 2003). Open reading frames (ORFs) are the major component of viral genomes. Our understanding of the composition of viral ORFs informs our understanding of the virus itself. A message RNA (mRNA) is transcribed from the viral genome and the host cell ribosomes then translate mRNAs into proteins. The proteins that form the virus particles, the enzymes that replicate the viral genomes, and the proteins that counter the host cell response to the intruder are all projected by the triplet codons that lie between the initiation AUG and the termination codon of the ORFs. Different genomic sequences can encode the same proteins, yet preferences exist for codons.

**FIGURE 1:**
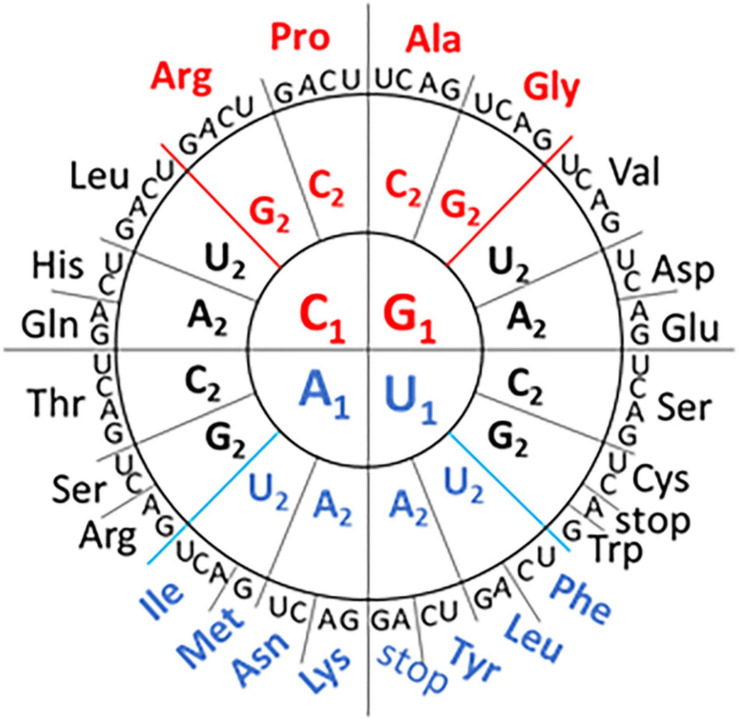
Codon Wheel adapted from Grosjean and Westhof (2016). The first nucleotide (N1) of the codon is displayed in the inner circle, the second nucleotide (N2) in the middle circle, and the third nucleotide (N3) in the outer circle with the encoded amino acid three letter code. GC-rich codons (in the first two positions) with “strong” network interactions are shown in red font and the AU-rich codons with “weak” network interactions are shown in blue font. The remaining codons are classified as having “intermediate” network interactions.

Redundancy in the genetic code means that different codons can fulfill the same function. However, there are biases in synonymous codon use that appear to be related to the GC content of the genome and the availability of host cell tRNAs to decode the message (Chevance et al., 2014; Kustin and Stern, 2021). Recent work by Grosjean and Westhof (2016) has mapped the network interactions linked to codon usage (Figure 1). The GC rich codons encode the earliest prebiotic amino acids while the more diverse amino acids associated with metabolic and modifying enzymes use the AU rich codons. While the codon grouping is based primarily on the GC or AU content at the first two codon positions, the grouping isn’t based on GC content alone. For example, the GGU (Gly) codon with “strong” network interactions has the same nucleotide content as the GUG (Val) and UGG (Trp) codons with “intermediate” network interactions (Figure 1). This interpretation of the genetic code has not previously been used to contribute to our understanding of influenza genomes.

Influenza viruses are segmented negative-strand RNA viruses that replicate in the host cell nucleus [reviewed in Dou et al. (2018)]. There are no DNA intermediates. A virus-encoded RNA-dependent RNA polymerase (RdRP) is associated with the end of each viral RNA (Murti et al., 1988). The influenza replicative machinery may recruit different co-factors to accomplish the production of different products (cRNA, gRNA, and mRNA) (Moncorge et al., 2010; Krischuns et al., 2021). The process of genomic replication is error prone (Cheung et al., 2014; Pauly et al., 2017). The low fidelity of RdRPs results in a high mutational burden in RNA viral genomes (Fitzsimmons et al., 2018). The composition of this population must be conserved enough to propagate the species but also diverse enough to include variants that can survive a range of selective pressures.

A virus must survive a range of selective pressures during its lifecycle: environmental conditions prior to encountering a new host, host immune responses, and competition for resources. Some selective pressures, such as those involving antigenic interactions, occur at the protein level (Wong et al., 2010). Others, such as the stability of genomic folding, are influenced by nucleic acid sequence (Atkinson et al., 2014; Phakaratsakul et al., 2018). Because viral genomes are mostly comprised of ORFs the robustness of the genome will depend on codon usage and the prevalence of codon-pairs plays a role in this. Deoptimizing codon or codon-pair usage has been used as a strategy to create attenuated viral vaccine strains (Le Nouen et al., 2019). Here we use various analyses of influenza genomes to reveal a trend in nucleotide content over time and a bias in codon-pair preference among influenza genomes. An awareness of this bias may help our understanding of virus evolution and inform the creation of codon deoptimized viral genomes.

## Methods

Influenza sequence data was obtained from different sources. Sequence source information for individual virus genomes is provided in Table 1. The relative synonymous codon usage (RSCU) and nucleotide composition was calculated for each genome using CAIcal (Puigbo et al., 2008). Results were exported to Microsoft Excel files to generate graphs.

**TABLE 1:** Influenza strain name and source location for sequences used for nucleotide and dinucleotide composition and RSCU analyses.

**Virus type**	**Strain name**	**Sequence source**
B ancestral	B/Lee/40	Influenza Research Database
B ancestral	B/Russia/69	Influenza Research Database
B Yamagata	B/Phuket/3073/2013	GISAID EPI_ISL_166957
B Victoria	B/Colorado/06/2017	NCBI (txid1987257)
H1N1 pandemic	A/Brevig Mission/1/1918	NCBI (txid88776)
H1N1 seasonal	A/New Jersey/11/1976	Influenza Research Database
H1N1 seasonal	A/New Caledonia/20/1999	GISAID EPI_ISL_22626
pdmH1N1 2009	A/California/7/2009	GISAID EPI_ISL_391380
H3N2	A/Aichi/2/1968	Influenza Research Database
H3N2	A/Brisbane/10/2007	Influenza Research Database
C	C/Ann Arbor/1/50	Influenza Research Database
C	C/Victoria/2/2012	Influenza Research Database

Codon usage tables (including codon-pair frequencies) were obtained from the High-performance Integrated Virtual Environment tables (Athey et al., 2017) for influenza A virus (NCBI:txid11320), influenza B virus (NCBI:txid11520), influenza C virus (NCBI:txid11552), three mammalian genomes (*Homo sapiens*, *Sus scrofa*, and Gyotis) and one avian genome (*Gallus gallus*). The codon-pairs present in each of the genomes were tabulated and the frequency calculated. Codon-pairs that were more frequent (greater than three standard deviations above the mean) or absent from the influenza genomes were identified. Each codon was characterized by type (“weak,” “strong,” or “intermediate”) based on network interactions shown in Figure 1 and the 4,096 possible codon- pairs were grouped as follows: weak:weak, 196 pairs; weak:intermediate, 868 pairs; intermediate:intermediate, 961 pairs; weak:strong, 448 pairs; strong:intermediate, 992 pairs; and strong:strong, 256 pairs. The 340 codon-pairs that included a termination codon were excluded from the analysis. The frequency of absent and overrepresented codon-pairs within the group was calculated.

## Results

Genome composition changes over time and evolutionary trends have been reported for different influenza viruses (Wong et al., 2010; Smith et al., 2018; Gu et al., 2019). We expand on our previous comparison of the nucleotide variant composition of two influenza B viruses (Plant et al., 2020a,b) and include influenza A and C viruses. The dinucleotide composition of three reference influenza genomes are compared with that of three mammalian genomes and one avian genome (Supplementary Figure 1). The dinucleotide composition of the three influenza genomes more closely resembled each other than the mammalian and avian genomes. The trend for higher AU content has previously been described for influenza and other RNA virus genomes (Kustin and Stern, 2021). Because the influenza genomes contain similar nucleotide composition and are subjected to similar evolutionary pressures, we next looked for genomic changes over time.

Here we compare the changes in influenza A, B, and C genomes (Table 1) isolated at different times over the last century. Influenza A H1N1 viruses have been isolated from humans infected from 1918 on (Taubenberger et al., 1997). Some influenza A lineages have arisen from zoonoses and circulated for several decades before being displaced by new lineages (Saunders-Hastings and Krewski, 2016). Influenza B viruses have been isolated from humans since the 1940s. In the 1970s the influenza B viruses split into two antigenically distinct lineages which have continued to co-circulate (Rota et al., 1990). Around the same time the influenza B lineage split occurred the influenza A H3N2 viruses emerged in the human population and have circulated since (Both et al., 1983). Influenza C viruses cause less disease than influenza A and B viruses, but sequence information is available from strains from the 1950s onward (Buonagurio et al., 1986). Representative influenza A, B, and C genomes isolated at different times over the last century were selected for further analysis (Table 1).

**FIGURE 2:**
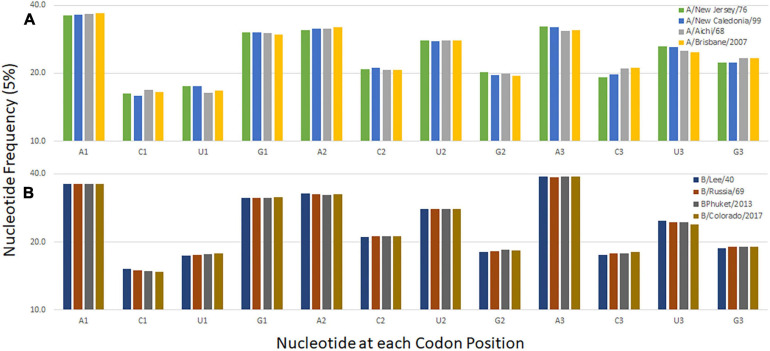
Codon nucleotide composition for influenza A **(A)** and influenza B **(B)** viruses. The frequency of each of the four nucleotides at each of the three codon positions is shown.

The nucleotide composition at the first, second, and third codon position of temporally distinct influenza B viruses was calculated (Figure 2B). A decrease in the percentage of C1 and U3 usage and increases in U1 and C3 usage are observed. Similar trends are seen when the same analysis performed for influenza A/H3N2 viruses and the seasonal A/H1N1 viruses A/New Jersey/11/1976 and A/New Caledonia/20/1999 (Figure 2A). The trend is not observed for the pandemic viruses A/Brevig Mission/1/1918 and A/California/7/2009 are included with the seasonal viruses but is apparent when the pandemic viruses are analyzed separately (Supplementary Figure 2). These viruses differ from the subsequent and preceding H1 viruses, respectively, because they are both zoonotic viruses with genomes adapted to the prior host species. The trend at the first codon position is also observed in the influenza C viruses (Supplementary Figure 2). The pressures driving adaptation to a new host species may differ from those that drive change in viruses (like influenza B and C) that have a sustained presence in one host species (Bhatt et al., 2013).

The similar trends in influenza A, B, and C genome composition prompted further comparison of the influenza genomes as a group. We looked to see if the trends in pyrimidine usage at the third codon position were reflected in changes to the relative synonymous codon usage (RSCU). A pyrimidine transition in the third codon position does not change the amino acid sequence (Figure 1). However, we did not observe any consistent trends in RSCU between influenza types (Supplementary Figure 3). This suggests that selective pressures that led to altered pyrimidine content at the third position are not constrained by host tRNA abundance. A similar conclusion was reached by other researchers in the analysis of bias in a wider selection of RNA viruses (Kustin and Stern, 2021). We next looked at codon-pair content in the three influenza genomes.

**FIGURE 3:**
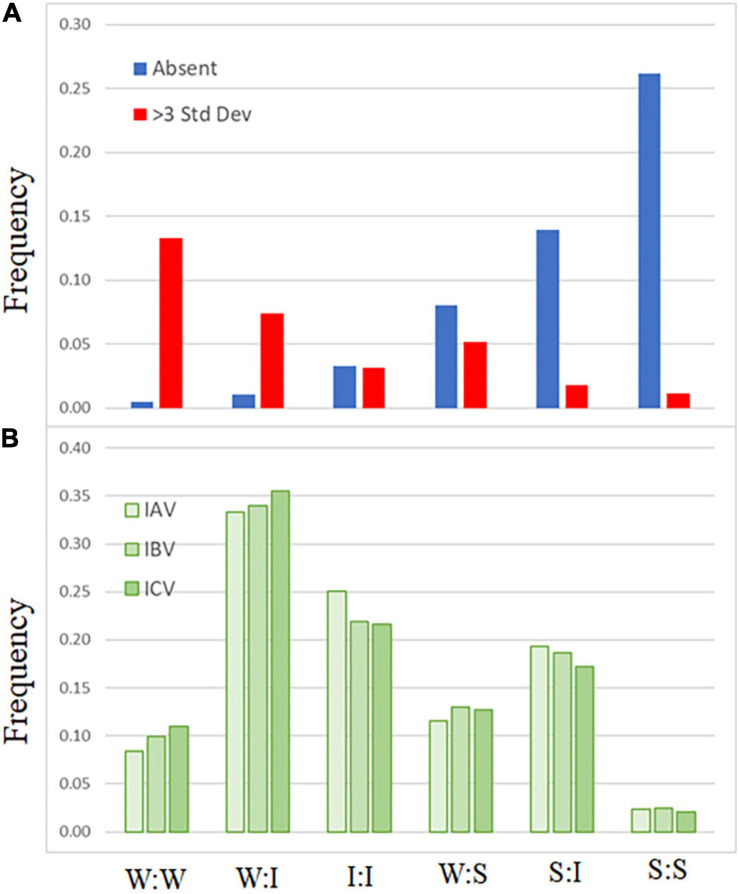
Frequency of influenza codon-pairs grouped by type. Codon-pairs from influenza A, B, and C genomes were grouped by network interaction strength [weak (W), intermediate (I), strong (S); see Figure 1]. The frequency of the codon-pairs that were absent or over-represented (>3 standard deviations above the mean frequency of all codon-pairs) was calculated for each group **(A)**. The expected frequency of the codon-pairs was calculated from the codon usage for influenza A, B, and C viruses **(B)**.

A comparison of codon-pair frequency among influenza A, B, and C genomes reveals a pattern in the codon-pairs that are more frequent or absent. The codon-pairs were grouped according to the strength of the network interaction (Figure 1). The majority of over-represented pairs present in all three influenza genomes are those with both codons having weak network interactions (Figure 3A). Conversely, the majority of codon-pairs missing in all three influenza types (excluding codon-pairs with termination codons) are those with strong network interactions for both codons. Thirteen percent (26 of the 196 possible) weak:weak codon-pairs are present in the influenza genomes at a frequency greater than three standard deviations above the mean (Figure 3A). Twenty-six percent (67 of the 256 possible) strong:strong codon-pairs are absent from the influenza genomes. These results indicate that codon-pairs with weak network interactions are preferable in influenza genomes.

## Discussion

The evolutionary robustness of a genome enables a virus to persist and avoid extinction. There are different characteristics of robust genomes and analogies may be made between different organisms (Mestek Boukhibar and Barkoulas, 2016). Here we describe a codon-pair bias common among three types of influenza virus and postulate that it facilitates virus survival. The dinucleotide content is similar among the three viral genomes (Supplementary Figure 1). For the viruses that have persisted in humans for longer periods of time (not recent zoonoses) there is also a trend in mononucleotide content in the third codon position (Figure 2). However, the trend for pyrimidine choice at this position does not translate into an obvious trend in the relative synonymous codon usage among the three influenza virus types. This suggests that the N3-N1 dinucleotide that borders the codon-pair has a role in influenza genome evolution as described by others (Gu et al., 2019). While investigating this trend we uncovered a bias in codon-pairs related to the strength of network interactions.

The better adapted a virus is to its host environment the better it will be able to compete with other viruses. Humans are the common host species for influenza A, B, and C viruses and other respiratory viruses. Competition among these viruses for the same resources will result in selection of viruses that replicate more efficiently. These selective pressures could affect both RNA transcription and protein production. At a molecular level we suggest that genomes dominated by codons with weak network interactions may have an advantage and that selection for these types of genomes may be observed in different viruses competing in the same host environment. It has been proposed that A-rich RNA genomes promote less secondary structure which would provide fewer targets for the eukaryotic immune system (Kustin and Stern, 2021). The same A-rich genome would also lead to fewer interactions between the mRNA and tRNA (Grosjean and Westhof, 2016). One study suggests that codon bias in influenza genomes may be associated with tRNA content during infection (Smith et al., 2018). But analysis of RSCU indicated that the speed of translation was not a likely cause of mutational bias in viral genomes (Kustin and Stern, 2021). These observations align with codon-pair bias we describe in this study.

A balance between replication speed and polymerase fidelity is required to maintain a viable genome (Fitzsimmons et al., 2018). The speed of transcription is dependent on the template and available substrates (Coggins et al., 2020). RNA structure has been implicated in reducing transcription speed (Zamft et al., 2012). It has been suggested that, because other nucleotides have more pairing options, A-rich viral genomes have less structure (Kustin and Stern, 2021). Additionally, it has been proposed that the pairing of codons fine tunes mRNA structure (Moura et al., 2007). The implication from these studies is that efficient replication of an RNA virus is better served by a genome with minimal structural requirements and our work links that to codon-pair preferences.

There are multiple pressures that affect virus genome composition and it is difficult to tease them apart. Influenza strains that have jumped from one host species to another are reported to evolve faster than strains that have circulated in the same host species during the same timeframe (Bhatt et al., 2013). In this work we have focused on the strains that have circulated in humans for extended periods of time and the results suggest that competition is affecting the rate of evolution. The H1N1 viruses re-entered the human population in 1976 and temporal changes in CG dinucleotides content reported since 1977 were not observed in the first half of the twentieth century (Gu et al., 2019). Because H1N1 viruses previously circulated in humans without change in CG content we suggest that the change in dinucleotide content reported by Gu et al., was due to a new selective pressure, such as competition from H3N2 strains. We hypothesize that the mononucleotide change and codon-pair preference described in herein derive from different selective pressures as the trend and bias was also observed in H3N2, influenza B and influenza C viruses during different time frames.

There is evidence demonstrating that some trends in virus nucleotide content are related to host cell responses. Low CG dinucleotide content has been reported in a wide range of viral genomes (Kunec and Osterrieder, 2016). Increasing the CG dinucleotide content in HIV genomes makes them more susceptible to RNA degradation by the ZAP enzyme (Takata et al., 2017). Suppression of genomic CpG content, including at the C3-G1 dinucleotide that borders codon-pairs, disproportionately reduces the number of codons with “strong” network interactions. However, when we calculate the expected frequency of codon-pairs, grouped by network interaction strength (Figure 3B), we still observe a bias in the frequency of over-represented weak:weak pairings and an absence of strong:strong codon-pairs (Figure 3A).

There are limitations to this study. Viral genomes are tightly packed with information including overlapping ORFs, replication and packaging signals (Li et al., 2021). These place constraints on different parts of the genome and, because of this, any analysis of the genome as a whole must be considered in this context. In our analyses we use only the coding regions of the genomes. The 5′ and 3′ non-coding portions of each segment of the genome are highly conserved where the RdRP interacts with these regions (Murti et al., 1988; Krischuns et al., 2021). Some regions of the segment contain packaging signals or are more densely associated with nucleoprotein (Li et al., 2021) and we did not analyze these separately. Future studies examining the relationship between codon-pair bias and specific coding regions will require the comparison of larger numbers of viruses. The changes in mononucleotide composition reported here are from just two temporally distinct strains (for influenza B, C, and influenza A/H1N1, and A/H3N2 strains). A more expansive analysis of strains spanning the timeframe for each influenza type is needed to improve the robustness of this analysis. Our analysis was limited to influenza virus genomes. More work is needed to determine if a bias related to network interactions is present in positive-strand RNA viruses, DNA viruses, or genomes of cellular organisms.

Here we have described a bias in codon-pairs in three types of influenza. A codon-pair bias identified by Kunec and Osterrieder (Kunec and Osterrieder, 2016) negatively correlated with GC content in RNA viruses but not DNA viruses. Codon grouping using network interactions has a relationship with GC content at the first two nucleotide positions but also groups codons with the same GC content separately (Figure 1). This feature might be useful in furthering our understanding of how different genomes evolve.

## Data Availability Statement

The original contributions presented in the study are included in the article/Supplementary Material, further inquiries can be directed to the corresponding author/s.

## Author Contributions

EP analyzed the data and drafted the manuscript. ZY provided supervision. Both authors contributed to the article and approved the submitted version.

## Conflict of Interest

The authors declare that the research was conducted in the absence of any commercial or financial relationships that could be construed as a potential conflict of interest.
