# Supplementary material for: A Codon-Pair Bias Associated With Network Interactions in Influenza A, B, and C Genomes
Source: Front Genet. 2021 Jul 6;12:699141. doi: 10.3389/fgene.2021.699141 (PMC8290168; doi:10.3389/fgene.2021.699141)
Supplement: Supplementary file 1 [file Data_Sheet_1.docx]

Supplementary Material

# Supplementary Figures


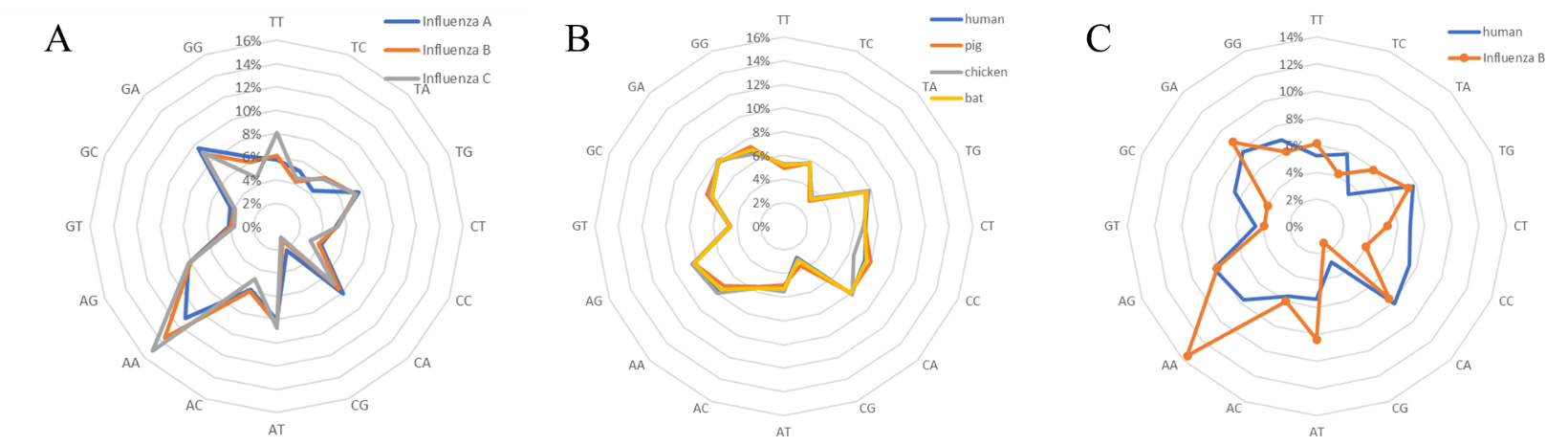


**Supplementary Figure 1a.** Dinucleotide composition among influenza genomes differs from host genome composition. Panel A shows the percentage of each dinucleotide present in the three reference influenza genomes from HIVE-CUT (reference 17 main text). Panel B shows the composition from human, pig, chicken and bat genomes. Panel C compares the dinucleotide composition of influenza B and human genomes.


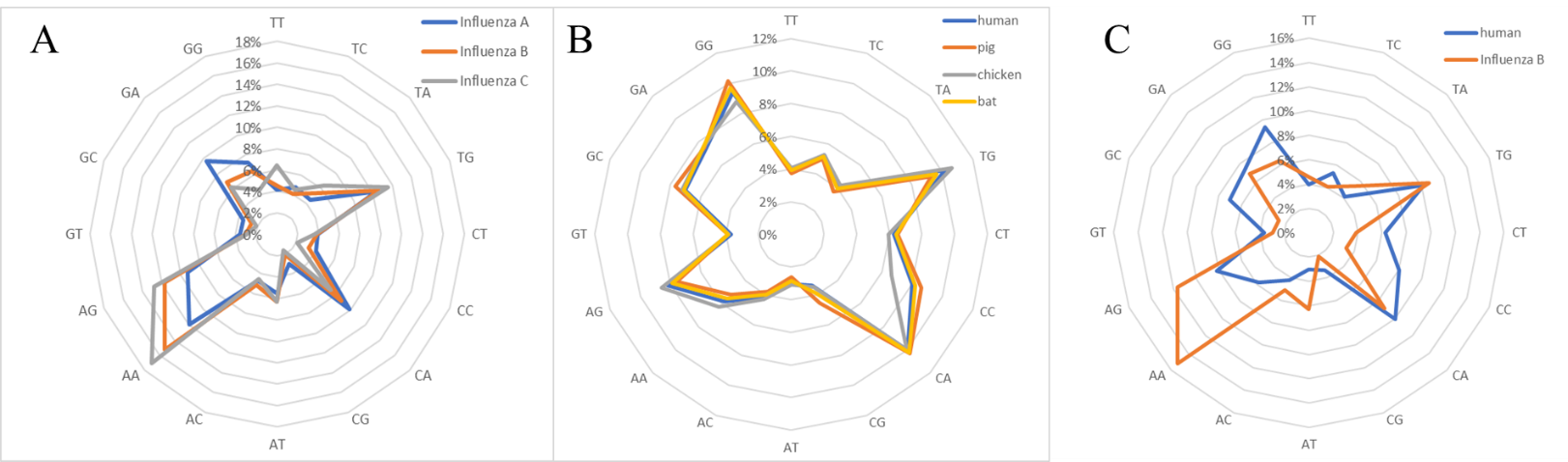


**Supplementary Figure 1b.** Influenza 3-1 junction dinucleotide frequency differs from host frequency. Panel A shows the percentage of each dinucleotide present at the codon junction in the three reference influenza genomes from HIVE-CUT(reference 17 main text). Panel B shows the composition from human, pig, chicken and bat genomes. Panel C compares the dinucleotide composition of influenza B and human genomes.


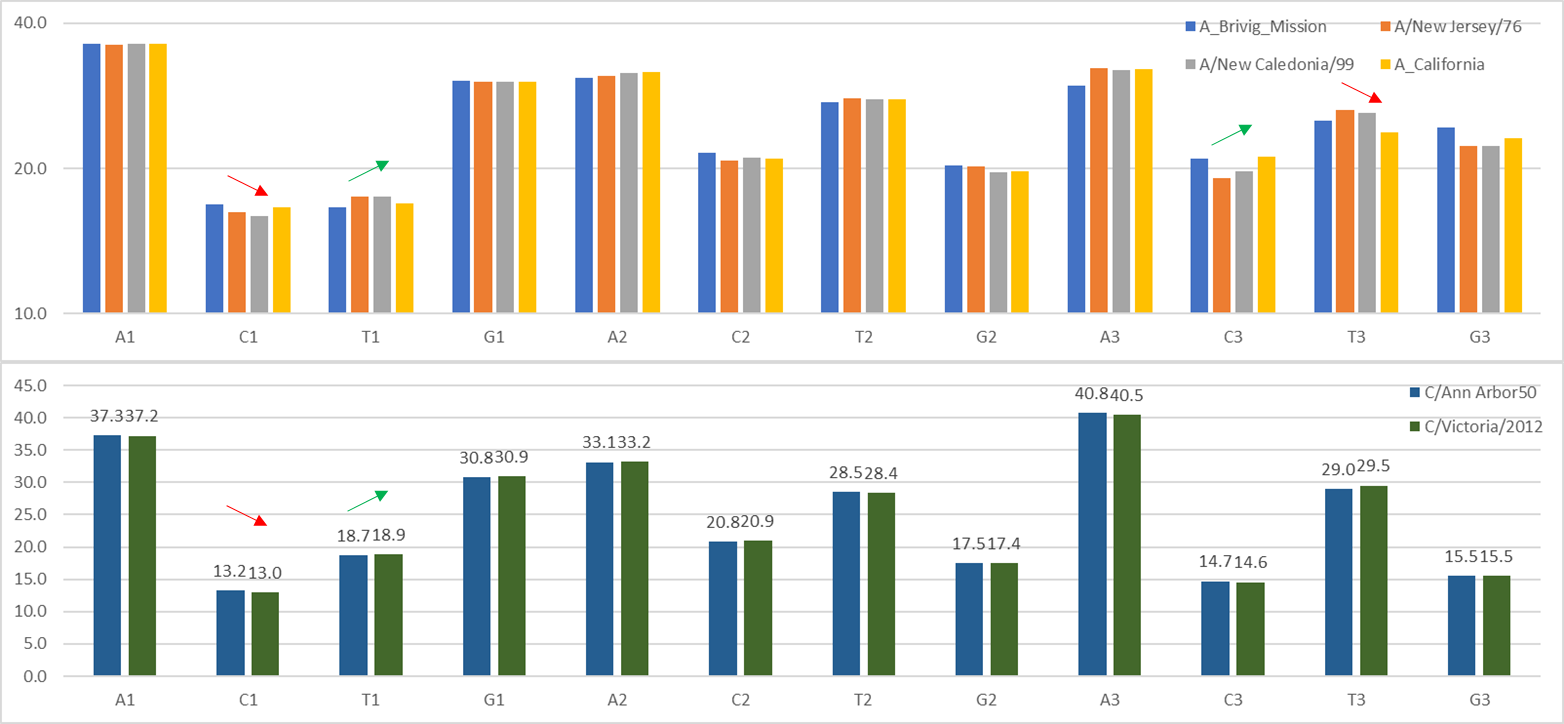


**Supplementary Figure 2a.** Changes in C/T usage in the first and third codon position across influenza virus types. Codon nucleotide composition for influenza A (H1N1) and influenza C viruses are shown. Strains are shown according to year of isolation. Two pandemic H1N1 strains flank two seasonal strains. Arrows are shown to highlight the trend and % composition is included for the influenza C graph. The % composition for the H1N1 viruses is included in supplementary figure 2b.


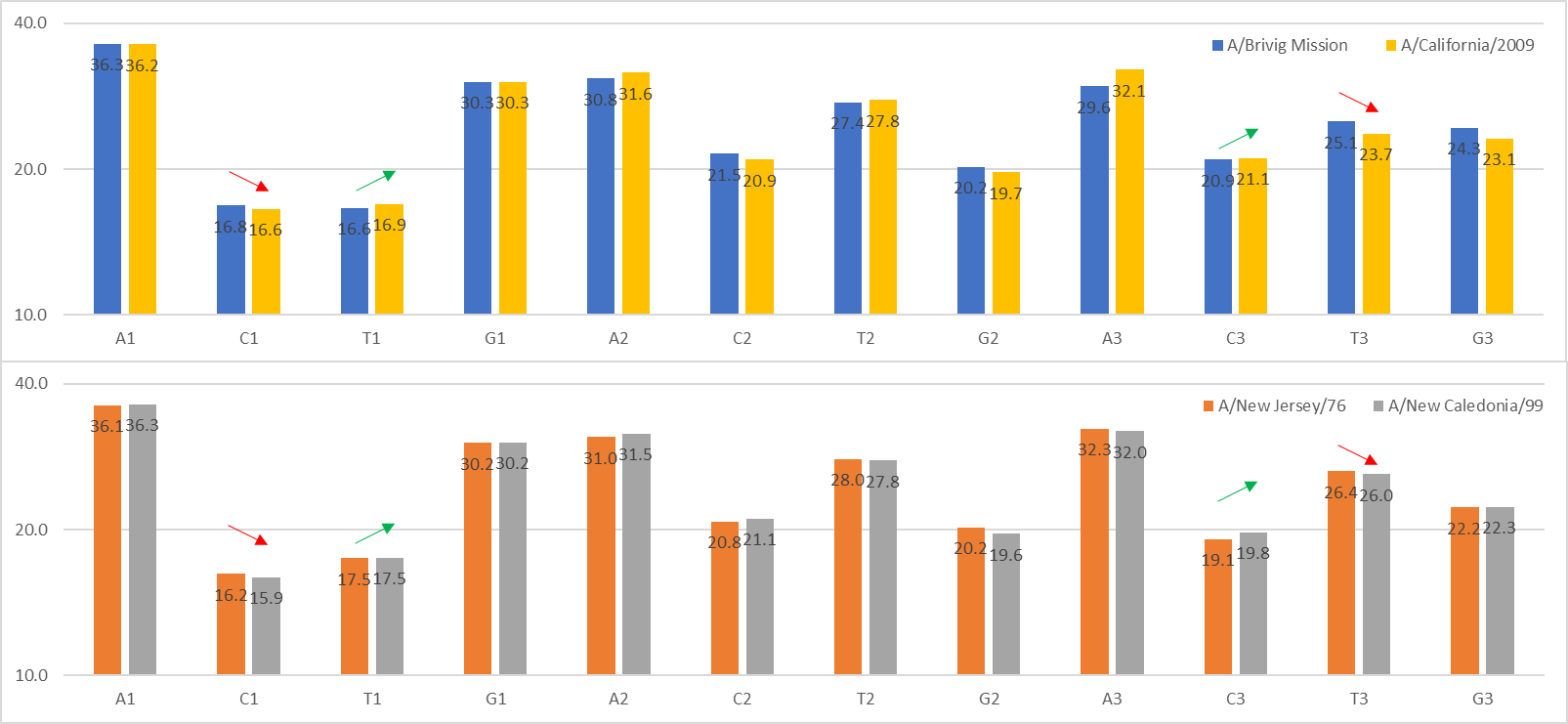


**Supplementary Figure 2b.** Changes in C/T usage in the first and third codon position for influenza A/H1N1 pandemic and seasonal viruses. Codon content is shown for two pandemic viruses in the upper panel and two seasonal in the lower panel. Arrows are shown to highlight the trend and bars are labelled with % content.


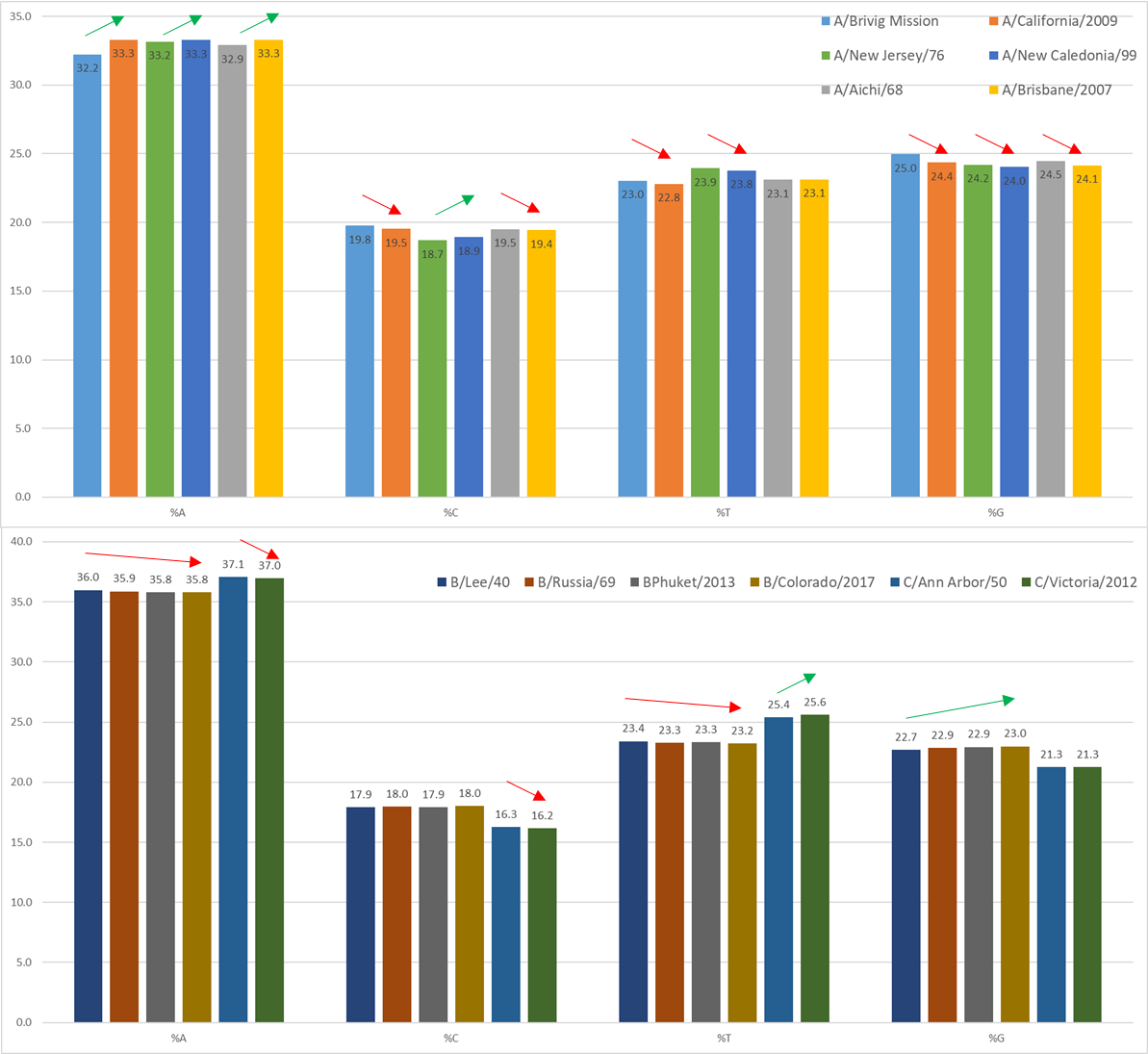


**Supplementary Figure 2c.** Variation and temporal changes in genomic nucleotide usage across influenza virus types. Nucleotide frequency is shown for pandemic A/H1N1, seasonal A/H1N1, A/H3N2, influenza B and influenza C virus types. Arrows are included to highlight temporal trends. Bars are labelled with % composition.

Note that there is variation in nucleotide content among influenza types (frequency scales differ between panels). Total adenosine content is higher in influenza B and C types than influenza A strains and show opposite trends (decrease/increase). The opposite trend is observed for guanosine content.

The total uracil content (%T) is most similar across types (22.8-25.6%), followed by cytosine (16.2-19.8%), guanosine (21.3-25.0%), with adenosine content most varied (32.2-37.1%).


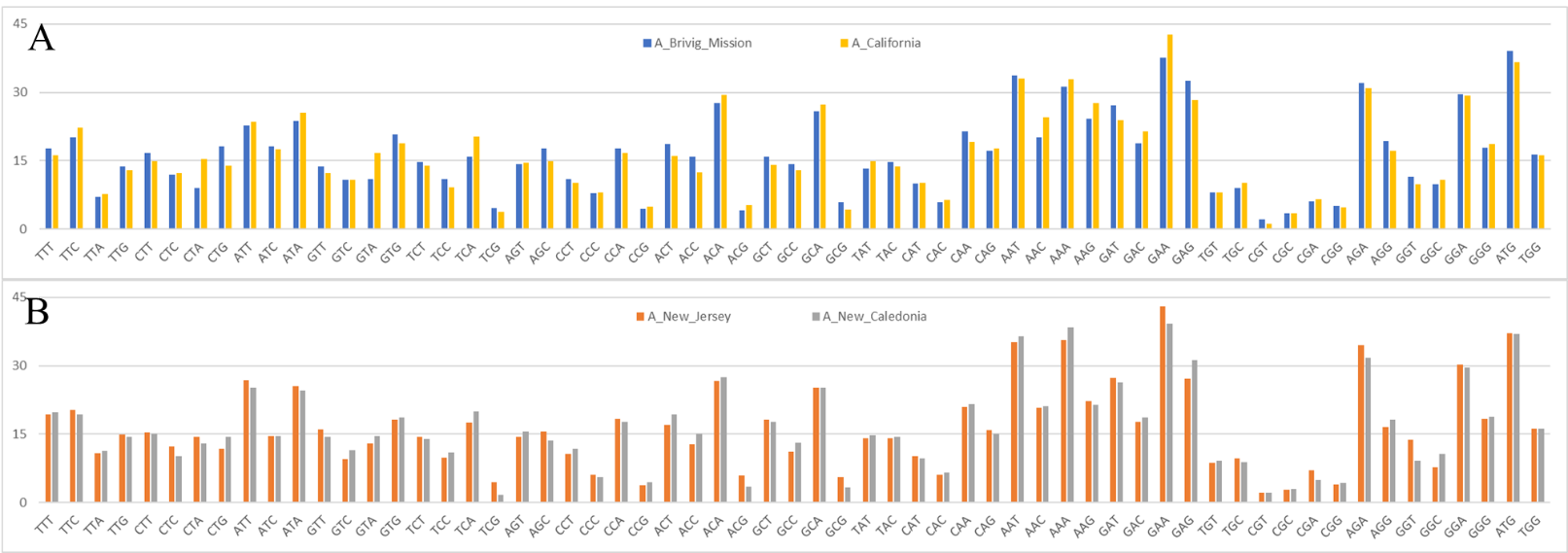


**Supplementary Figure 3a.** Trends in Relative Synonymous Codon Usage. The RSCU is shown for influenza A viruses: panel A, pandemic H1N1; and panel B, seasonal H1N1. Note that for codons ending in a pyrimidine the increase or decrease between temporally separate strains in one panel is not the same in the other.


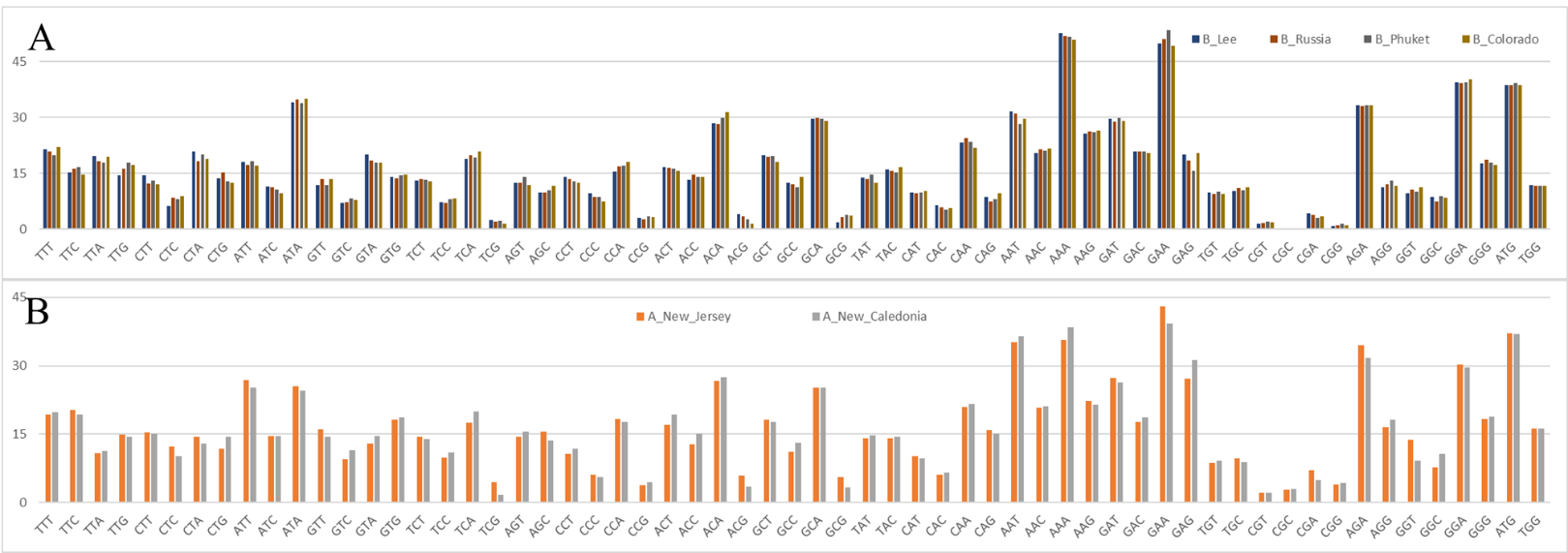


**Supplementary Figure 3b.** Trends in Relative Synonymous Codon Usage. The RSCU is shown for influenza B and seasonal A/H1N1 strains. Although a temporal trend for increased C3 and decreased T3 was observed for influenza B strains (Figure 2 main text) that pattern is not observed in consistently across the RSCU groups. An increase or decrease in pyrimidine use in panel A (influenza B viruses) is not consistently matched in the panel B (seasonal A/H1N1 viruses).


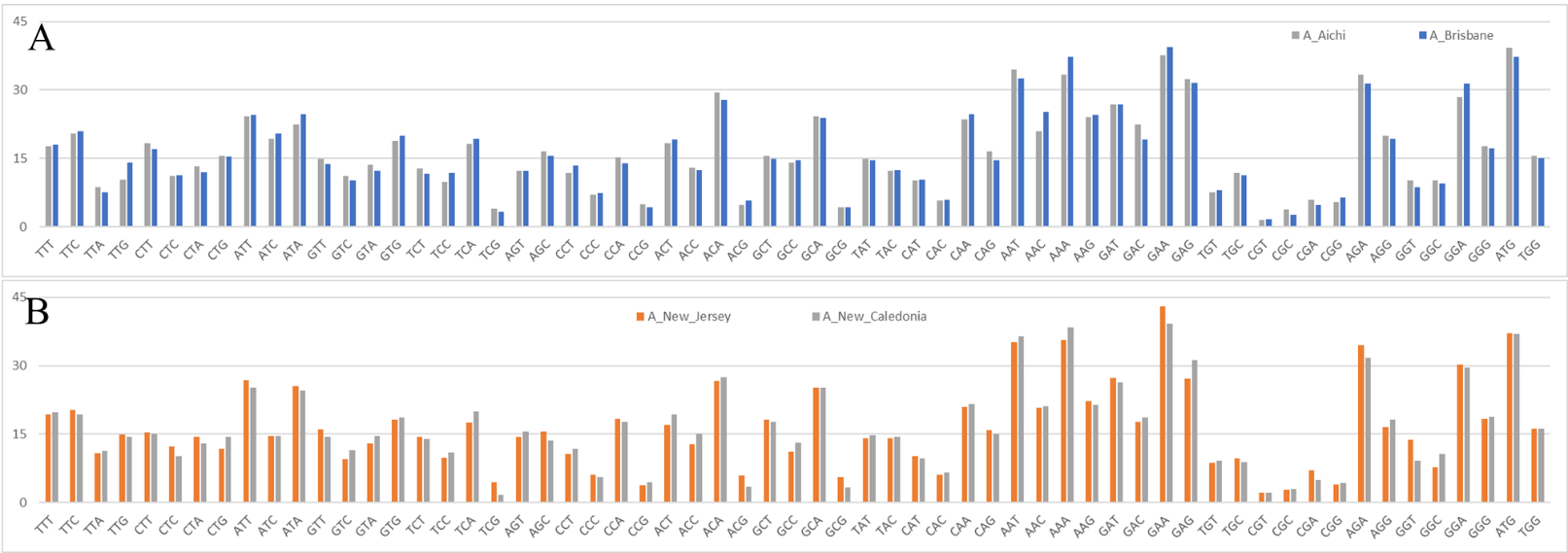


**Supplementary Figure 3c.** Trends in Relative Synonymous Codon Usage. The RSCU is shown for influenza A viruses: panel A, H3N2; and panel B, seasonal H1N1. Note that for codons ending in a pyrimidine the increase or decrease between temporally separate strains in one panel is not the same in the other.


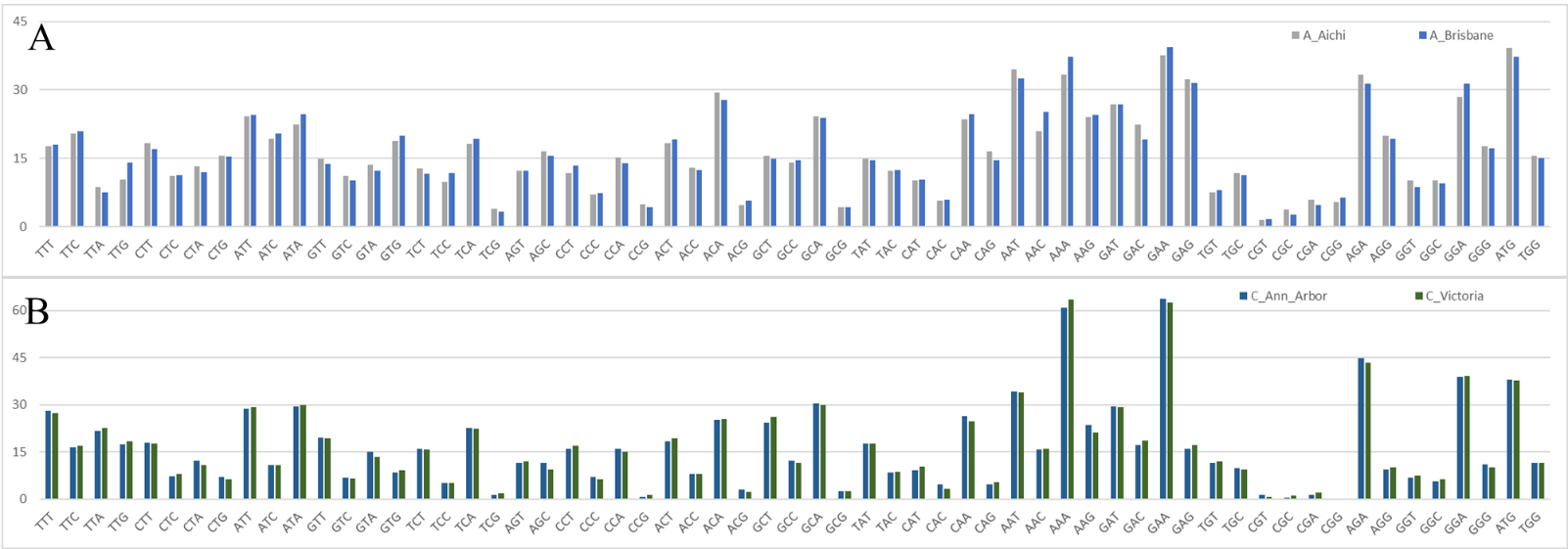


**Supplementary Figure 3d.** Trends in Relative Synonymous Codon Usage. The RSCU is shown for influenza viruses: panel A, A/H3N2; and panel B, influenza C. Note that influenza C viruses utilize fewer codons ending in a cytosine. An increase in C3 was observed for other influenza viruses but not influenza C (Supplementary Figure 2).
